# Comparison of Intraocular Lens Formulas in Patients With Postoperative Refractive Surprise

**DOI:** 10.7759/cureus.74991

**Published:** 2024-12-02

**Authors:** Karine D Bojikian, Dana Lee, Sarah Lee, Marlow Schulz, Andrew Chen, Philip Chen

**Affiliations:** 1 Ophthalmology, University of Washington, Seattle, USA

**Keywords:** intraocular lens, intraocular lens power calculation formulas, ocular biometry, phacoemulsification cataract surgery, refractive surprise

## Abstract

Objective: This study investigates the refractive accuracy of eight intraocular lens (IOL) power calculation formulas in patients with postoperative refractive surprise after phacoemulsification. It aims to determine if a different formula could result in better refractive outcomes in these eyes.

Methods and analysis: We retrospectively reviewed consecutive patients undergoing uncomplicated phacoemulsification as a sole procedure between March 2007 and September 2020 at the University of Washington by glaucoma subspecialists as part of a study investigating cataract surgery in normal eyes. The refractive surprise was defined as > 0.5 D difference between the zeroed-out predicted error (PE) using the Barrett Universal II (BUII) formula and postoperative refraction at four and 12 weeks. Mean absolute error (MAE) and zeroed out PE for Hoffer Q, Holladay 1 and 2, Sanders Retzlaff Kraff theoretical (SRK/T), radial basis function (RBF) 3.0, Kane, and Ladas super formula AI (LSFAI) formulas were calculated.

Results: Of 440 eyes, 88 (20.0%) met inclusion criteria (MAE 0.77 ± 0.28). Of these, 35.2% and 64.8% had myopic (MAE 0.76 ± 0.23) and hyperopic (0.78 ± 0.31 D) surprise, respectively. The predicted SE significantly differed from postoperative refraction for all formulas (p* *≤ 0.035). The proportion of eyes with refractive surprise was not different comparing BUII with RBF, Kane, and LSFAI (range 19.5 to 20.2%; p ≥ 0.831), but was significantly higher for Holladay I, Holladay II, Hoffer Q, and SRK/T (29.5-31.8%; p < 0.001). For 13 eyes (3.0%) with refractive surprise ≥ 1.0 D (MAE 1.34 ± 0.25), no formula reduced the MAE below 1.22 D. Logistic regression revealed shorter axial length (AL) to be a risk factor for both myopic and hyperopic refractive surprise; total astigmatism and biometric ratios (keratometry(K)/AL and anterior chamber depth(ACD)/AL) were novel risk factors for hyperopic surprise.

Conclusion: Four more recent IOL calculation formulas were statistically equivalent in accuracy, indicating that these eyes are outliers across different formulas. Shorter eyes had a higher risk for refractive surprise. Novel biometric parameters warrant further investigation to improve refractive outcomes.

## Introduction

Phacoemulsification remains one of the most common surgeries in the United States [1]. Implanting the most accurate intraocular lens (IOL) power for an individual undergoing cataract surgery is necessary to achieve the desired refraction and, ultimately, patient satisfaction. Intraocular lens power calculations are determined through mathematical equations utilizing unique ocular data obtained from the individual's eyes, including the corneal curvature, the axial length (AL), and anterior chamber depth (ACD, measured from corneal epithelium to lens). For many years, ophthalmologists calculated IOL power with three classic vergence-based formulas (Hoffer Q, Holladay 1, and SRK/T), but many other formulas have been developed in the past decade.

Previously, researchers have investigated the accuracy of IOL power calculations compared to corneal curvature [2], AL [3], IOL type, gender, and even race [4]. Some authors have reported that Barrett Universal II (BUII) results in the least number of refractive surprises [5]. Others have shown that Kane results in the least refractive error when investigating eyes with AL greater than 26 mm [6]. Pereira et al. [7] evaluated the accuracy of 12 different IOL power formulas and demonstrated that Kane had the highest overall accuracy, while Holladay 1 and BUII had higher accuracy in short and long AL cases, respectively. With the advent of the application of AI in medicine, machine learning has emerged for use in IOL calculations. The original Ladas super formula combined multiple formulas to enhance accuracy. It has now been transformed into a data-driven method, namely the Ladas super formula AI (LSFAI) [8].

Despite several prior studies on determining the ideal formula to calculate IOL power in different populations, a gap remains in understanding whether some formulas would have been superior to others in patients with a significant postoperative (PO) refractive surprise. In this study, we investigate the refractive predictability and accuracy of eight IOL power calculation formulas, including older and newer generation formulas, in a series of normal patients without other ocular diseases who had uncomplicated phacoemulsification and had PO refractive surprise > 0.5 diopters (D). We aimed to assess whether any of the formulas could have predicted the refractive surprise outcome.

## Materials and methods

The Ethics Committee of the Human Subjects Division of the University of Washington approved this study (approval no. STUDY00011008), and informed consent was deemed unnecessary due to the retrospective nature of this research. This study followed the tenets of the Declaration of Helsinki and was conducted in compliance with the Health Insurance Portability and Accountability Act. We retrospectively reviewed consecutive patients undergoing uncomplicated phacoemulsification as a sole procedure between March 2007 and September 2020 at the University of Washington by glaucoma subspecialists, as part of a study investigating cataract surgery in normal eyes (eyes without eye diseases other than cataracts, including age-related macular degeneration, diabetic retinopathy, epiretinal membrane, keratoconus, and other cornea dystrophies, among others) and eyes with glaucoma. Exclusion criteria included: 1) primary surgeon was an ophthalmology resident physician; 2) any visually significant eye diagnosis other than cataracts; 3) history of refractive surgery; 4) less than 12 weeks of PO follow-up; and 5) need for additional surgery within 12 weeks from initial cataract surgery. When both eyes of one patient were eligible, the eye undergoing surgery first was chosen for the study.

For the entire cohort, we recorded pertinent clinical information before phacoemulsification, including baseline demographics, ophthalmic biometry obtained by IOL Master 500 (Carl Zeiss Meditec Inc., Jenna, DEU), preoperative and PO spherical equivalent (SE), and preoperative intraocular pressure. All Snellen visual acuity measurements were converted to the logarithm of the minimum angle of resolution (logMAR) values for statistical analysis.

Cataract surgery was performed using topical plus intracameral anesthesia and standard phacoemulsification technique with temporal clear-corneal incision. Pupil expansion techniques and devices were used to enlarge the pupil as necessary, and incisions that were not watertight were sutured. The power of the implanted IOL was chosen at the surgeon's discretion. Surgically induced astigmatism was assumed to be zero for all.

The SE predictions were performed by the authors using different resources. For the Hoffer Q, Holladay 1, Holladay 2, and the Sanders Retzlaff Kraff theoretical (SRK/T) formulas, the Holladay IOL Consultant version 2023.0322 (Holladay Consulting Inc., Bellaire, TX, USA) was used. For BUII, Kane, and radial basis function (RBF) 3.0 formulas, the European Society of Cataract & Refractive Surgeons (ESRCS) online calculator was used (https://iolcalculator.escrs.org). For the LSFAI, the online calculator (https://iolcalc.com) was used. The standard (manufacturer) optical A-constant was utilized based on the implanted IOL as follows: Sofport® L161AO: 118.7 (Bausch & Lomb, Vaughan, ON, CAN); AcrySof® Multipiece MA60AC: 119.2 (Alcon Laboratories Inc., Fort Worth, TX, USA); AcrySof® Expand® MA60MA: 118.9; AcrySof® SA60AT: 118.8; AcrySof® IQ SN60WF: 119.1; Tecnis® ZCB00: 119.4 (Johnson & Johnson Surgical Vision, Jacksonville, FL, USA). If an IOL did not have a listed A constant in the Kane online calculator, the eye was excluded. Surgically induced astigmatism was not included in the calculations because no IOL power calculation formula requires notation of it since it should not affect postoperative spherical equivalent refractive error.

We zeroed out the arithmetic mean predicted error (PE) by adjusting the refractive PE for each eye up or down by an amount equal to the arithmetic mean error in that group. [9] Irrespective of any stated target in the chart, the new predicted SE by BUII for the IOL implanted was used as the point of reference for this study. The refraction lane was 6 m. The refractive surprise was defined as the difference between PO refraction within four and 12 weeks after surgery and the BUII new predicted SE (i.e., zeroed out PE). Patients with refractive surprise > 0.5 D were included in the analysis. We calculated the mean absolute error (MAE) for each formula. The MAE is the mean absolute zeroed-out PE value. 

Statistical analyses were performed using SPSS Statistics version 29.0.1.0 (IBM Corp., Armonk, NY, USA). Univariate analysis was performed using a paired two-tailed t-test for continuous variables and Pearson chi-square for categorical variables. Friedmann’s test (Friedmann ANOVA) was used to compare the differences in absolute error among the different formulas. A Tukey test for pairwise mean comparisons was used for comparing PEs given the possibility of non-normal data distribution. A p-value < 0.05 was considered significant. The McNemar test was used for comparing the proportions of eyes with refractive surprise using BUII vs. the other seven formulas. Binary logistic regression was used to determine factors associated with myopic and hyperopic refractive surprise.

## Results

Of the 440 normal patients who underwent uncomplicated cataract surgery between 2007 and 2020, a total of 88 eyes (20.0%) had a refractive surprise > 0.5 D (Table 1). Thirty-one (35.2%) had a myopic refractive surprise (MAE 0.76 ± 0.23 D), and 57 eyes (64.8%) had a hyperopic surprise (MAE 0.78 ± 0.31 D). Keratometry readings, AL, and ACD were not different between eyes with and without refractive surprise, but astigmatism was greater in eyes with refractive surprise.

**Table 1:** Baseline demographics and preoperative and postoperative descriptive statistics for eyes without refractive surprise > ±0.5 (n=352) and for eyes with refractive surprise > ±0.5 diopters (n=88). All values are percentages or mean ± standard deviation, or median (in square brackets). LogMAR: Logarithm of the minimum angle of resolution, D: Diopters

Parameters	Eyes without refractive surprise (n=352)	Eyes with refractive surprise (n=88)	p-value (*t-test; ¥=Pearson chi-square)
Age (years)	68.6 ± 9.7 (range 29 to 92)	68.8 ± 9.4 (range 32 to 88)	0.859*
Sex			
Female	200 (56.8%)	50 (56.8%)	0.547¥
Male	152 (43.2%)	38 (43.2%)	
Race			
Caucasian	238 (67.6%)	59 (67.0%)	0.851¥
Asian	56 (7.7%)	13 (14.8%)	
African American	27 (7.7%)	8 (9.1%)	
Other	31 (8.8%)	8 (9.1%)	
Preoperative visual acuity (LogMAR)	0.263 ± 0.335	0.431 ± 1.435	0.279*
Preoperative spherical equivalent (D)	-1.6 ± 3.8 [-0.6]	-1.8 ± 3.6 [-0.5]	0.661*
Axial length (mm)	24.2 ± 1.6	24.1 ± 1.3	0.413*
Axial length (mm)			
<22	10 (2.8%)	3 (3.4%)	0.481¥
≥22 and < 26	298 (84.7%)	78 (88.6%)	
≥ 26	44 (12.5%)	7 (8.0%)	
Anterior chamber depth (mm)	3.2 ± 0.4	3.1 ± 0.4	0.177*
Preoperative keratometry reading K1 (D)	43.5 ± 1.6 [43.5]	43.5 ± 1.7 [43.3]	0.886*
Preoperative reratometry reading K2 (D)	44.4 ± 1.6 [44.2]	44.7 ± 2.0 [44.4]	0.230*
Preoperative intraocular pressure (mmHg)	15.3 ± 2.9	15.4 ± 2.6	0.827*
Type of intraocular lens			
Tecnis® ZCBOO (Johnson & Johnson Surgical Vision, Jacksonville, FL, USA)	146 (41.5%)	37 (42.1%)	0.955¥
Sofport® LI61AO (Bausch & Lomb, Vaughan, ON, CAN)	153 (43.4%)	38 (43.1%)	
Acrysof® MA60AC (Alcon Laboratories Inc., Fort Worth, TX, USA)	28 (8.0%)	8 (9.1%)	
Acrysof® SA60AT	25 (7.1%)	5 (5.7%)	
Intraocular lens power (D)	19.4 ± 4.4 [20.0]	19.6 ± 4.1 [20.0]	0.576*
Postoperative visual acuity (LogMAR)	0.011 ± 0.083	0.048 ± 0.131	0.012*
Postoperative spherical equivalent (D)	-0.4 ± 0.6 [-0.3]	-0.4 ± 0.9 [-0.2]	0.651*
Refractive surprise (D)			
Myopic >0.50 and <1.00	N/A	27 (30.7%)	
Myopic ≥ 1.00		4 (4.5%)	
Hyperopic>0.50 and <1.00		48 (54.5%)	
Hyperopic ≥ 1.00		9 (10.2%)	

Among the 440 included eyes, the percentage of eyes with ≤ 0.5 D and < 1.0 D of absolute zeroed-out PE were as follows: BUII 80.0% and 97.0%; RBF 3.0 80.5% and 96.4%; Kane 79.8% and 95.5%; LSFAI 79.8% and 96.1%; Holladay 1 70.6% and 92.9%; Hoffer Q 69.5% and 93.2%; SRK/T 69.5% and 90.9%; and Holladay 2 68.2% and 90.7%, respectively. Compared with BUII, no difference in the proportion of eyes with refractive surprise of ≤ 0.5 and < 1.0 D was seen with RBF (p = 0.831 and 0.375, respectively), Kane (p = 1.00 and 0.065), or LSFAI (p = 1.0 and 0.289). But the Holladay I (p < 0.001 and 0.035), Hoffer Q (p < 0.001 and 0.003), SRK/T (p < 0.001 and <0.001), and Holladay II (p < 0.001 and < 0.001) formulas had significantly higher proportions of patients with refractive surprise at those levels (McNemar test).

The mean absolute error ranged from 0.71 (LSFAI) to 0.85 (Holladay 2) D in the entire refractive surprise cohort. It ranged from 0.72 (Kane) to 1.01 (Holladay 2) D in the myopic surprise subgroup and from 0.64 (LSF AI) to 0.78 (Holladay 2) D in the hyperopic subgroup (Table 2). The PO refractive outcome was significantly different from the predicted SE for all eight IOL power formulas in the entire refractive surprise cohort and in the myopic and hyperopic surprise cohorts (p ≤ 0.035) (Table 2).

**Table 2:** Baseline ocular measurements and the MAE of zeroed-out refractive outcome for the intraocular lens calculation formulas in 88 patients with refractive surprise. Values are presented as mean ± standard deviation, and median and range. *A p-value <0.05 compared with BUII MAE: Mean absolute error, BUII: Barrett universal II, SE: Spherical equivalent, D: Diopters, RBF: Radial basis function; LSFAI: Ladas super formula AI

Parameters	All refractive surprise (n=88)	MAE (D)	p-value ¶ = Tukey test for pairwise mean comparisons	All myopic surprise (n=31)	MAE (D)	p-value ¶ = Tukey test for pairwise mean comparisons	All hyperopic surprise (n=57)	MAE (D)	p-value ¶ = Tukey test for pairwise mean comparisons
Axial length (mm)	24.08 ± 1.28 [24.03; 21.49 to 26.82]			23.54 ± 1.21 [23.50; 21.49 to 26.14]			24.37 ± 1.23 [24.35; 22.00 to 26.82]		
Anterior chamber depth (mm)	3.09 ± 0.44 [3.07; 1.92 to 4.02]			3.12 ± 0.43 [3.06; 2.44 to 4.02]			3.08 ± 0.45 [3.07; 1.92 to 3.94]		
Postoperative SE	-0.37 ± 0.93 [-0.19; -3.50 to 1.00]			-1.34 ± 0.60 [-1.25; -3.50 to -0.25]			0.17 ± 0.58 [0.25; -1.75 to 1.00]		
Barrett predicted SE	-0.64 ± 0.60 [-0.57; -2.63 to 0.74]	0.77	0.017	-0.46 ± 0.57 [-0.48; -2.53 to 0.74]	0.76	<0.001	-0.74 ± 0.59 [-0.63; -2.63 to 0.37]	0.78	<0.001
Hoffer Q predicted SE	-0.55 ± 0.58 [-0.46; -2.58 to 0.75]	0.77	0.033	-0.42 ± 0.64 [-0.33; -2.58 to 0.75]	0.86	<0.001	-0.62 ± 0.55 [-0.47; -2.54 to 0.51]	0.72	<0.001
Kane predicted SE	-0.63 ± 0.58 [-0.56; -2.62 to 0.80]	0.73	0.024	-0.48 ± 0.59 [-0.38; -2.62 to 0.80]	0.72	<0.001	-0.72 ± 0.56 [-0.60; -2.52 to 0.25]	0.74	<0.001
LSFAI predicted SE	-0.51 ± 0.59 [-0.44; -2.36 to 1.02]	0.71*	0.035	-0.35 ± 0.56 [-0.32; -2.32 to 1.02]	0.86	<0.001	-0.60 ± 0.59 [-0.48; -2.36 to 0.46]	0.64*	<0.001
RBF 3.0 predicted SE	-0.62 ± 0.57 [-0.54; -2.52 to 0.83]	0.75	0.006	-0.47 ± 0.57 [-0.48; -2.52 to 0.83]	0.75	<0.001	-0.70 ± 0.55 [-0.60; -2.50 to 0.33]	0.75	<0.001
SRK/T predicted SE	-0.47 ± 0.55 [-0.37; -2.50 to 0.74]	0.81	0.004	-0.33 ± 0.54 [-0.30; -2.46 to 0.74]	1.00	<0.001	-0.55 ± 0.55 [-0.49; -2.50 to 0.40]	0.71	<0.001
Holladay 1 predicted SE	-0.49 ± 0.54 [-0.42; -2.37 to 0.74]	0.78	0.010	-0.38 ± 0.56 [-0.31; -2.37 to 0.74]	0.94	<0.001	-0.55 ± 0.52 [-0.46; -2.19 to 0.46]	0.69*	<0.001
Holladay 2 predicted SE	-0.52 ± 0.57 [-0.47; -2.52 to 0.95]	0.85*	0.027	-0.34 ± 0.60 [-0.22; -2.52 to 0.95]	1.01	<0.001	-0.62 ± 0.52 [-0.58; -2.31 to 0.35]	0.78	<0.001

Friedman’s test (Friedman’s ANOVA) revealed significant differences in the zeroed-out PE among the eight IOL power formulas in the entire cohort and for the subgroups of myopic and hyperopic surprise (p < 0.001 for all). Post hoc analysis with Wilcoxon signed-rank test with Bonferroni correction for multiple comparisons using all 88 eyes revealed a statistically significant difference in zeroed-out absolute errors between BUII and LSFAI and Holladay 2 (p < 0.001 for both), but not between BUII and the other formulas (p ≥ 0.080) (Table 2). For the 31 myopic surprise eyes, there were no significant differences between BUII and the other formulas (p ≥ 0.189). For the 57 eyes with hyperopic surprise, LSFAI and Holladay I had a lower MAE than BUII (p ≤ 0.04) while the other formulas were comparable to BUII (p ≥ 0.078) (Table 2).

For 13 eyes (3.0%) with a refractive surprise ≥1.0 D, the MAE was 1.34 ± 0.25 D and ranged from 1.22 ± 0.44 D (Hoffer Q) to 1.31 ± 0.28 D (Holladay 2) (data not shown). For hyperopic eyes with a refractive surprise ≥1.0 D (n=9) there were significant differences in the zeroed-out absolute PE between BUII and LSFAI, Holladay 1 and SRK/T (p ≤ 0.021, Friedman’s test), but no formula reduced the MAE to < 1.0 D; for myopic eyes (n=4) no significant difference was found between zeroed-out absolute PE between BUII and all formulas (p ≥ 0.066).

We examined factors associated with refractive surprise using binary logistic regression, using the entire cohort (n=440). We elected to analyze only our results with BUII, given the equivalence between BUII and the other more recent formulas. We included age, sex, race, preoperative visual acuity, AL, anterior chamber depth (ACD), average keratometry, astigmatism, ratio of ACD/AL, ratio of average keratometry/axial length (K/AL), and intraocular pressure (IOP) difference (postoperative IOP at four weeks minus preoperative IOP) as covariates. Factors associated with myopia > 0.5D included shorter AL (p = 0.006) and positive IOP difference (p = 0.035). Factors associated with hyperopia > 0.5D included higher astigmatism (p = 0.012), higher average keratometry reading (p = 0.013), steeper K/AL (p = 0.015), shallower ACD/AL (p = 0.016), and shorter AL (p = 0.021) (Table 3).

**Table 3:** Risk factors for refractive surprise with BUII formula (n=440) B: Regression coefficient, ACD: Anterior chamber depth, AL: Axial length, K: Keratometry, D: Diopters

Parameters	B	Standard error	p-value	Odds ratio	95% confidence interval
Myopia >0.5D (n=31)					
Axial length per mm	-0.414	0.150	0.006	0.661	0.493, 0.886
IOP difference per mmHg	0.125	0.059	0.035	1.133	1.009, 1.273
Hyperopia >0.5D (n=57)					
Astigmatism per D	0.494	0.196	0.012	1.639	1.117, 2.407
Average keratometry per D	1.825	0.733	0.013	6.205	1.48, 26.1
Average K/AL per 0.1D	-4.618	1.786	0.015	0.010	0.000, 0.327
ACD/AL per 0.1mm ACD	-2.77	0.964	0.016	0.063	0.009, 0.413
Axial length per mm	-3.186	1.305	0.021	0.041	0.003, 0.534

## Discussion

The accuracy of IOL power calculation depends on accurate preoperative biometric measurements, the difference between the predicted and achieved effective lens position, and possible PO fluctuations in corneal power [10,11]. Due to advances in the methods of ocular biometry and an abundance of theoretical, ray-tracing, and AI-based IOL power calculation formulas, postoperative SE predictions have improved to around 75% to 80% within ± 0.5 D of the target in uncomplicated eyes without prior refractive surgery [12-16]. However, in a population of 440 normal eyes that underwent uncomplicated phacoemulsification, our data show that for the 20% of patients where the target refractive outcome was missed by more than 0.5D, the overall refractive outcome for those patients was not clinically different using zeroed-out results of four more recent IOL power calculation formulas. Approximately 3% of eyes had ≥ 1.0D of refractive surprise with these four formulas, and none had clinically different performance. Notably, the proportion of eyes with > 0.5D or ≥ 1.0D of refractive surprise was significantly worse for four older formulas. Our data could guide cataract surgeons and may help set expectations for patients. 

The different IOL power formulas have attempted to improve the accuracy of their refractive outcome predictions, predominantly by increasing the number of variables they assess. Third-generation formulas (Holladay 1, SRK/T, and Hoffer Q) incorporated the effect of corneal curvature, while fourth-generation formulas such as the Holladay 2 and BUII formulas use other factors, such as corneal diameter (CD) and lens thickness (LT) [11]. Artificial intelligence-based formulas such as LSFAI and RBF 3.0 have other optional inputs, such as central corneal thickness. In our study, we used the standard (manufacturer) A-constants for the implanted IOL for each formula calculation. Our MAE ranged from 0.71 (LSFAI) to 0.85 (Holladay) D for the entire cohort and from 0.72 (Kane) to 1.01 (Holladay 2) and 0.63 (LSFAI) to 0.78 (Holladay 2) D in the subgroups of myopic and hyperopic surprise, respectively. We found that the postoperative refractive outcome was significantly different from the predicted SE for all eight IOL power formulas (i.e., no improved outcomes in this set of problematic eyes). Additionally, we found no clinically important differences in zeroed-out absolute PE between BUII and the other formulas; LSFAI would have resulted in the lowest MAE by 0.06D, while Holladay 2 would have resulted in the highest MAE by 0.08D.

Shorter AL is known to be a risk factor for ametropia after phacoemulsification [17], and binomial logistic regression of our study population confirmed that finding. In addition, myopic refractive surprise was associated with an increase in postoperative IOP. Some authors have found that IOP reduction after phacoemulsification in normal eyes is associated with a significant reduction in AL, enough to affect refraction by approximately 0.23 to 0.3D in an eye with average AL, keratometry, and ACD [18,19]. Intuitively, an increase in IOP could lead to greater myopia; unfortunately, this is a risk factor that cannot be accurately predicted prior to surgery. The hyperopic refractive surprise was also associated with greater astigmatism, higher average keratometry, a smaller ratio of K/AL, and a smaller ratio of ACD/AL. Steeper keratometry readings are known to have a greater influence on IOL calculation than other corneal parameters, although the BUII has been reported to generally perform well with variation in keratometry [12,20,21]. However, for all significant risk factors for hyperopic surprise other than astigmatism, the 95% confidence intervals were very large, likely due to the small sample size.

Our study has several limitations. The retrospective nature of our study might have resulted in errors related to the non-standardization of treatments and methods, incompleteness of data recording, and variability of PO regimens and follow-up. Refractive errors are not only due to the inaccuracy of the IOL power calculation formula but can also arise from inaccurate biometry measurements, refraction variability or inaccuracy, and ocular surface issues, including dry eyes, and in a retrospective study, we are unable to control all these factors. Our results are based on IOL Master 500 biometry and only include AL, keratometry, ACD, and white-to-white measurements. We did not include data on LT or corneal central thickness measurements, which might have improved refractive predictions, though some authors have reported that the addition of LT data did not improve the accuracy of IOL calculations with BUII, Kane, or RBF 3.0 formulas [22].

As our surgeon cohort was comprised of glaucoma subspecialists, and we excluded patients with other ocular comorbidities to reduce confounders, our sample size is relatively small, with consequent limitations on analysis. Refractions were performed by multiple practitioners, including ophthalmic technicians, optometrists, and ophthalmologists, though all were experienced in refraction. Additionally, we used standard (manufacturer) A-constants, and individualized optimization of A-constants could have improved overall results. Still, 80.0% and 97.0% of eyes in our total cohort were within 0.5D and 1.0D, respectively, of zeroed-out predicted refractive outcome with BUII, which is consistent with recent reports of cataract surgery outcomes [5,12,14-16].

## Conclusions

In this cohort of normal patients without other ocular disease, 20% of patients had refractive surprise > 0.5D and 3% had refractive surprise ≥ 1.0D; four newer generation formulas were performed equally, and none would have improved the overall accuracy of IOL calculation. Shorter AL was a risk factor for both myopic and hyperopic refractive surprise with BUII. Total astigmatism and ratios of K/AL and ACD/AL may be candidates for further research as potential predictors for refractive outcomes. Further research evaluating other biometric variables that may contribute to effective lens position is needed.
